# Use of Thrombopoietin Receptor Agonists in Childhood Immune Thrombocytopenia

**DOI:** 10.3389/fped.2015.00070

**Published:** 2015-08-13

**Authors:** Angelica Maria Garzon, William Beau Mitchell

**Affiliations:** ^1^Department of Pediatrics, Memorial Sloan Kettering Cancer Center, New York, NY, USA; ^2^Laboratory of Platelet Biology, New York Blood Center, New York, NY, USA; ^3^Division of Pediatric Hematology Oncology, Weill Cornell Medical College, New York, NY, USA

**Keywords:** immune thrombocytopenia, chronic immune thrombocytopenia, thrombopoietin receptor agonists, eltrombopag, romiplostim

## Abstract

Most children with immune thrombocytopenia (ITP) will have spontaneous remission regardless of therapy, while about 20% will go on to have chronic ITP. In those children with chronic ITP who need treatment, standard therapies for acute ITP may have adverse effects that complicate their long-term use. Thus, alternative treatment options are needed for children with chronic ITP. Thrombopoietin receptor agonists (TPO-RA) have been shown to be safe and efficacious in adults with ITP, and represent a new treatment option for children with chronic ITP. One TPO-RA, eltrombopag, is now approved for children. Clinical trials in children are ongoing and data are emerging on safety and efficacy. This review will focus on the physiology of TPO-RA, their clinical use in children, as well as the long-term safety issues that need to be considered when using these agents.

## Introduction

Immune thrombocytopenia (ITP) is an acquired autoimmune disorder that affects both children and adults, resulting in isolated platelet counts <100 × 10^9^/L and potentially life-threatening bleeding. ITP is generally the result of increased platelet destruction and decreased platelet production leading to thrombocytopenia. IgG antibodies bind to platelet membrane glycoproteins, typically GPIIb/IIIa, GPIb/IX, or GPIa/IIa, causing phagocytosis by the reticuloendothelial system in the liver and spleen, resulting in early clearance, and hence a decreased platelet life span (1). Impaired production of platelets also contributes to ITP. The antibodies bind to the surface of megakaryocytes (which express the same glycoproteins) in the bone marrow leading to destruction of megakaryocytes and decreased platelet production (2). ITP is a diagnosis of exclusion and is either primary or secondary. Primary ITP occurs in the absence of any inciting cause. Secondary ITP is caused by an underlying disease or drug exposure. ITP is also classified based on duration. Patients have newly diagnosed ITP in the first 0–3 months, persistent ITP in the 3- to 12-month period, and chronic ITP at >12 months. ITP may also be classified on severity, which is determined based on the presence or absence of bleeding symptoms (3).

Approximately 80% of pediatric patients will respond to a single treatment or combination of first line therapies, which include watchful waiting, corticosteroids, IVIG, or anti-D (4, 5). For the remaining patients, additional second line treatments are needed. These generally include further immune suppression, for example, with anti-CD20 (rituximab), with the goal of decreasing the production of anti-platelet antibodies. Splenectomy is avoided in young children both because of the risk of bacterial sepsis and because children have a high rate of spontaneous resolution of ITP. A recently developed class of drug, which will be the focus of this review, is the thrombopoietin receptor agonists (TPO-RA). These agents target the megakaryocytes and drive them to increased platelet production.

## Thrombopoietin Receptor Agonists

Thrombopoietin (TPO) is a growth factor produced primarily by the liver that mediates its effects through the TPO receptor (cMPL). It is the most important growth factor for platelet production (6, 7). TPO binding to the cMPL receptor results in activation of the STAT and MAPK signaling pathways, and leads to megakaryocyte differentiation and growth. Its circulating concentration is regulated primarily by the platelet and megakaryocyte mass, and is usually increased when platelets are decreased, such as in aplastic anemia. However, although platelets are decreased in ITP patients, endogenous TPO levels have been noted to be normal or even low in response to the platelet decrease (7, 8). This has been attributed to the increased megakaryocyte mass seen in the bone marrow of ITP patients.

The two first-generation TPO-RA were recombinant forms of human TPO (9). One was a full-length TPO protein produced in Chinese hamster ovary cells, and the other was a partial-length protein coupled to polyethylene glycol produced in *Escherichia coli*. These agents were targeted primarily at chemotherapy-induced thrombocytopenia. They had mixed success in patients receiving non-myeloablative chemotherapy. However, their use was discontinued after subjects developed auto-antibodies that cross reacted with endogenous TPO, leading to prolonged thrombocytopenia in some subjects.

**Figure 1:**
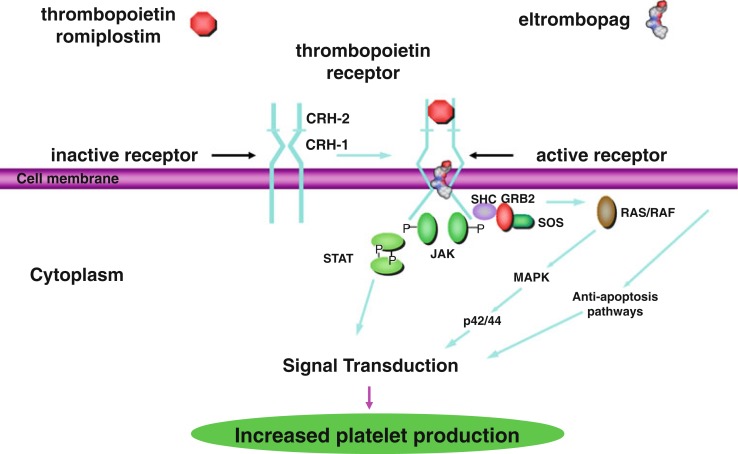
**Thrombopoietin receptor activation by thrombopoietin, romiplostim, and eltrombopag via the STAT/MAPK pathways for increased platelet production**. This research was originally published in International Journal of Hematology [Kuter (9) by The Japanese Society of Hematology].

The second generation thrombopoietic agents were designed to have no amino acid sequence homology to endogenous TPO and are structurally different from TPO to avoid development of neutralizing antibodies. These agents bind to and activate the TPO receptor, so are also TPO-RA (Figure 1) (9). Currently, there are two FDA approved second generation agents for adults, romiplostim and eltrombopag. As of June 11, 2015, eltrombopag received FDA approval for use in children >6 years of age. Romiplostim continues to be in trials for children.

Romiplostim (AMG 531, AMP-2, Nplate) was approved in 2008 by the FDA for use in adults with ITP. It is a peptibody consisting of two short peptides that are coupled to an immunoglobulin Fc domain, produced by recombinant DNA technology in *E. coli* (7). The peptides have no homology to TPO. Like TPO, romiplostim promotes megakaryocyte growth by binding to the TPO binding site on cMPL, resulting in activation of the STAT and MAPK signaling pathways, driving megakaryocyte proliferation and differentiation (Figure 1) (10). The peptides bind competitively to the TPO binding site, while the Fc domain is essential for increased half-life of the drug. Romiplostim is administered weekly subcutaneously at doses of 1–10 μg/kg.

Eltrombopag (SB497115, Promacta) was also approved in 2008 by the FDA for adults and 2015 for children >6 years of age with ITP not responsive to first line therapies. It is a small non-peptide agonist that binds to the transmembrane domain of cMPL, rather than the TPO binding site. Eltrombopag binding results in signaling through the STAT and MAPK signaling pathways, similar to the signaling mechanism of TPO, and promotes the growth of TPO-dependent cell lines (Figure 1) (11). Because eltrombopag does not bind to the same site of the TPO receptor as TPO, this non-competitive binding is thought to allow eltrombopag and TPO to have additive cell-signaling effects. Eltrombopag is administered orally daily at doses of up to 75 mg/day.

## Experience in Pediatrics

### Romiplostim

In 2011, Bussel et al. reported the first multicenter phase I–II randomized double-blind study of the use of a TPO-RA, romiplostim, in order to determine both safety and efficacy in children (12). The study enrolled 22 children aged 1–18 who had been diagnosed with ITP at least 6 months prior. Seventeen patients received romiplostim, starting at a dose of 1 μg/kg/week and escalated to 10 μg/kg/week, and five received placebo weekly for 12 weeks, with a platelet goal of 50–250 × 10^9^/L. To maintain this goal, the weekly median dose at 12 weeks was 5 μg/kg. Eighty-eight percent of patients achieved a platelet count ≥50 × 10^9^/L for two consecutive weeks, while patients in the placebo group had no effect. Platelet counts ≥50 × 10^9^/L were maintained for a median of 7 weeks in romiplostim patients and 0 week in placebo patients. In this group of patients, the most commonly reported adverse events were headache and epistaxis, and there were no serious adverse events.

That same year Elalfy et al., in Egypt, published a randomized placebo-controlled study of romiplostim in 18 children aged 2.5–6 years of age with chronic poorly responsive ITP (13). These patients had a median baseline count of 10.5 × 10^9^/L and were randomized (2:1) to receive romiplostim or placebo for 12 weeks. Romiplostim dosing began at 1 μg/kg/week and escalated to 5 μg/kg/week. The median weekly dose of romiplostim was 2 μg/kg. The platelet count goal of 50 × 10^9^/L was reached and maintained by 83% of patients receiving romiplostim. Fifty percent of patients reported at least one adverse event including headache, epistaxis, vomiting, and coughing, but there were no serious adverse events.

The following year a prospective study, also from Egypt, by Mokhtar et al., described romiplostim therapy in eight non-splenectomized children aged 3.4–15.2 years with unresponsive chronic ITP, although one patient was initially excluded because of grade 3 bone marrow reticulin (14). These children had ITP lasting 13 months to 7.3 years with a median of 2.4 years. Romiplostim dosing was initiated at 1 μg/kg/week and escalated by 1 μg/kg/week with a platelet count goal >50 × 10^9^/L. The duration of therapy varied between 1 and 22 weeks. Fifty-seven percent of patients demonstrated variable responses but then showed a rapid increase in platelet count when pulse steroid therapy was added. Adverse events were mild and transient.

In 2012, a general hospital in Madrid, Spain published a retrospective, longitudinal observational study of three pediatric patients refractory to treatment who were treated with romiplostim (15). Among the three patients, one was newly diagnosed and two had chronic ITP. These patients were started at a dose of 1 μg/kg/week and escalated up to 10 μg/kg/week. They were followed for 27–39 weeks. Responses were seen in 7–28 days, and were maintained for 37–91% of visits. The adverse events were headache and asthenia in one patient and mucocutaneous bleeding in another patient after stopping romiplostim. There were no serious adverse events.

In 2014, a retrospective national study in France from the CEREVANCE group, evaluated the use of romiplostim in 10 children aged 1–18 with non-responsive or refractory chronic ITP (16). Patients started romiplostim at an initial dose of 1 μg/kg/week and escalated up to 10 μg/kg/week with a platelet count goal of at least 50 × 10^9^/L. Median dose was between 4 and 10 μg/kg/week. The median duration of treatment was 9 months (range 3–36). Five children improved clinically and had disappearance of mucosal bleeding. The other five patients had no response and had persistent severe bleeding. Six children reported adverse events including local pain, headache, asthenia, abdominal pain, and one mood disorder, but no serious adverse events were seen.

In 2014, Seidel et al. in Austria described their center’s experience with romiplostim in treating seven patients between the ages of 2 and 17 with chronic ITP (17). These patients were started on a dose of 2–5 μg/kg/week and escalated to 10 μg/kg/week. Response rates were variable, but patients were noted to have increased platelet counts when combined with either IVIG, anti-D, mycophenolate mofetil, or rapamycin. This group reported no adverse events and had normal bone marrow evaluations.

Also, in 2014, Ramaswamy et al. published a retrospective analysis of 33 children aged 19 months to 19 years with ITP >6 months who received either romiplostim or eltrombopag (18). Of those 33 children, 21 received romiplostim and 12 received eltrombopag. The median starting dose was 5.0 μg/kg/week for romiplostim and 50 mg daily for eltrombopag. The mean maximum dose of romiplostim was 8.1 μg/kg/week and 75 mg daily for eltrombopag. Twenty-seven (82%) patients responded to TPO-RA, 18 of 21 to romiplostim, and 9 of 12 to eltrombopag. These 27 patients had platelet counts ≥50 × 10^9^/L and ≥20 × 10^9^/L above baseline for 2 consecutive weeks; 26 had 50% of platelet counts ≥50 × 10^9^/L. Duration of romiplostim use ranged from 6 to 44 months (11/18 ongoing) and of eltrombopag 23 to 53 months (7/12 ongoing). One patient on eltrombopag experienced a deep-vein thrombosis at the site of and ankle fracture but no other serious adverse events occurred. Among 24 bone marrows performed, 10 were after more than 2 years of therapy, 23 were normal and 1 was MF-2.

Most recently, in 2015, Bussel et al. published the results for the long-term use of romiplostim in children <18 years of age with chronic ITP (19). This patient cohort had completed the romiplostim phase I–II study. Twenty patients from the cohort continued on romiplostim for up to 109 weeks. All of them achieved platelet counts >50 × 10^9^/L. A subset of these, 12 patients, continued in a second extension study for up to 127 weeks. Overall, in this cohort, treatment duration reached a median of 167 weeks and the median dose was 5.4 μg/kg.

Overall, these studies have indicated that romiplostim has both good efficacy and safety in children with persistent or chronic ITP. The response rate has been 50–88%, similar to that seen in adults. The effective dosing is also similar to that seen in adults. Safety data have accumulated for several years of treatment and the side effects have been minor.

### Eltrombopag

Thus far, there are fewer published studies on the use of eltrombopag in children. However, at the 56th annual 2014 American Society of Hematology meeting, pooled data were presented from two studies (1450 PETIT and PETIT2) of children aged 1 to <18. Both of these studies were randomized, double-blind, and placebo-controlled study. Patients were randomized 2:1 to eltrombopag or placebo. After the placebo-controlled randomized phase, patients were eligible to complete 17 or 24 weeks of treatment with eltrombopag. Dose was adjusted based on platelet counts to a maximum of 75 mg daily.

A total of 174 subjects were enrolled in both studies. One hundred fifty-nine patients were randomized and 157 received ≥1 dose of randomized study treatment. During the randomized period 62% of children on eltrombopag versus 24% of children on placebo achieved a response with platelet counts ≥50 × 10^9^/L at least once between 1 and 6 weeks. At each week, a higher proportion of the eltrombopag group had a response versus placebo group. Thirteen percent of children on eltrombopag received rescue treatment compared to 31% of children on placebo. During the extension study, sustained reduction or discontinuation of baseline ITP medications was achieved by 50% of patients; 81% of patients had a platelet count response at least once; 52% had a platelet count response for ≥50% of assessments; and 38% responded for ≥75% of assessments. For weeks 13–24, 47% of subjects achieved responses.

The most common AEs were headache, upper respiratory tract infection, and nasopharyngitis. Serious AEs were reported in 8% of eltrombopag group versus 12% of placebo group. In the randomized period, an ALT elevation of three times ULN occurred in five patients receiving eltrombopag and none in the placebo group. In the extension study, there were an additional seven patients with ALT elevation to three times ULN. All ALT abnormalities resolved either while still on treatment or at discontinuation. No thromboembolic events were reported. Cataracts occurred in two patients; both had used corticosteroids and one had pre-existing cataracts.

These two large placebo-controlled studies indicate that eltrombopag has both good efficacy and a good safety profile in children, which led to its approval for children >6 years of age this year. Elevation of ALT was noted as an AE in some patients on the treatment arm. This distinguishes eltrombopag from romiplostim, which has not been reported to be associated with increased liver enzymes. The occurrence of cataracts in two study subjects is also unique to eltrombopag. Therefore, these potential AEs should be monitored when using eltrombopag.

## TPO-RA in Other Thrombocytopenias

TPO-RA have also been used for other causes of thrombocytopenia. Currently, eltrombopag is FDA approved for the treatment of thrombocytopenia in chronic Hepatitis C, and most recently received approval for the treatment of severe aplastic anemia. Eltrombopag has also been reported to increase platelet counts in patients with Wiskott–Aldrich syndrome and other inherited thrombocytopenias. Romiplostim is currently in trials for the treatment of thrombocytopenia other than ITP. Of note, while TPO-RA are not intended to be curative therapy and platelet counts will predictably fall after discontinuation, there have been reports of durable remission in adults after prolonged treatment with TPO-RA. This has yet to be reported in children.

## Dosing

In adults, the typical maintenance dose of romiplostim ranges between 3 and 8 mcg/kg for a target platelet count >50 × 10^9^/L (20). In children, a similar maintenance dose seems to be required. Median adult dosing of eltrombopag is 50 mg/day, as described in the EXTEND dose study (21). However, in children, it appears that higher dosing per weight may be needed but the recommended starting dose is 50 mg/day not to exceed 75 mg/day. The efficacy of both drugs is similar to those seen in adults with response rates of 80% and higher if transient responses are included.

## Safety and Monitoring

The most common side effects reported by both children and adults for both agents include headache, nausea, and vomiting. More significant adverse effects include arterial and venous thromboembolic events and bone marrow reticulin deposition/fibrosis with both agents and liver toxicity seen with eltrombopag. Arterial and venous thromboembolic events in adults have mostly occurred in patients with prior history of thrombosis (22). In children, there has been one reported thromboembolic event: one DVT in the setting of an ankle fracture (18). Bone marrow reticulin deposition is seen in both adults and children. In adults, approximately one-fifth of patients will develop a grade 2/3 myelofibrosis (23). This appears to be reversible once the TPO-RA is discontinued (22, 23). In children, reticulin deposition occurs less frequently: Ramaswamy et al. reported 23/24 normal bone marrows in pediatric patients, some after years on therapy, and Seidel reported 5/5 normal marrows after long-term use of romiplostim. Eltrombopag may cause elevated liver enzymes. In the patients receiving eltrombopag in the 6-week study by Bussel et al., in the RAISE study and in the EXTEND study, 10% of treatment group patients had liver enzyme (ALT) elevations to three times the upper limit of normal, while 3% in the placebo group had ALT elevations (24). The ALT returned to normal in some patients even while they continued on eltrombopag. In other patients, the ALT returned to normal after discontinuation of the study drug. Similar effects have been noted in children, as described in the PETIT trials.

Prior to starting either medication, patients should have baseline lab values for complete blood counts (CBCs) and liver function tests (AST, ALT, bilirubin), and should be monitored regularly. They should have CBCs to assess platelet counts weekly until a stable count is achieved and then counts can be monitored monthly. For patients taking eltrombopag, LFTs should be monitored weekly, followed by every other week during dose adjusting, and then monthly once a stable dose has been reached. More frequent monitoring for any abnormality is at the physician’s discretion. Patients on either agent should have their marrow examined periodically for reticulin. Although there are no consensus guidelines regarding frequency of monitoring the bone marrow for reticulin deposition, the authors usually examine the marrow annually.

## Conclusion

Ultimately, the goal of therapy in ITP is to increase the platelet count and prevent clinically significant bleeding. Thus far, TPO-RA have demonstrated response rates in children comparable to that of IVIG, and have been well tolerated with minor side effects. TPO-RA represent a paradigm shift in treatment of ITP in that they directly target a mechanism of thrombocytopenia with few off-target effects. The TPO-RA are not immunosuppressive and thus avoid the side effects of traditional immunosuppressive therapies. The most concerning side effects that are seen in adults, bone marrow reticulin deposition and thrombosis, have been less commonly seen in children. Long-term effects remain to be determined, but some children have already been in current clinical trials for several years (Table 1). Thus, for children who fail first line therapy, TPO-RA represent a novel and effective alternate therapy.

**Table 1:** **Current TPO-RA under study for use in ITP**.

**(a) Romiplostim and eltrombopag in children**	
NCT02201290	A long-term safety study of eltrombopag in pediatric patients with chronic ITP
NCT01880047	Safety and efficacy of eltrombopag at escalated doses
NCT01957176	A rollover study to provide continued treatment with eltrombopag
NCT01971684	Treatment decisions and outcomes in pediatric refractory ITP
NCT01071954	A study evaluating the safety and efficacy of long-term dosing of romiplostim
NCT02279173	Single arm, open-label, long-term study of romiplostim
NCT01971684	Treatment decisions and outcomes in pediatric refractory ITP
**(b) Other TPO-RA**	
Small molecules	
NCT01438840	Avatrombopag (E5501, AKR-501, YM477, AS 1670542): oral
NCT00621894	LGD-4665: oral
N/A	Totrombopag (SB-559448): oral
Recombinant human TPO	
NCT02139501	TPIAO: oral, licensed in China

## Author Contributions

AG wrote the paper. WM wrote the paper. Neither AG nor WM are PI’s or co-PI’s of any of the cited studies. WM works at an institution where several pediatric studies of TPO-RA are ongoing.

## Conflict of Interest Statement

The authors declare that the research was conducted in the absence of any commercial or financial relationships that could be construed as a potential conflict of interest.

## References

[1] Labarque V Van Geet C. Clinical practice: immune thrombocytopenia in paediatrics. Eur J Pediatr (2014) 173(2):163–72. 10.1007/s00431-013-2254-6 24390128

[2] Takahashi R Sekine N Nakatake T. Influence of monoclonal antiplatelet glycoprotein antibodies on in vitro human megakaryocyte colony formation and proplatelet formation. Blood (1999) 93(6):1951–8. 10068668

[3] Provan D Stasi R Newland A Blanchette V Bolton-Maggs P Bussel J International consensus report on the investigation and management of primary immune thrombocytopenia. Blood (2010) 115(2):168–86. 10.1182/blood-2009-06-225565 19846889

[4] Evim M Baytan B Günes A. Childhood immune thrombocytopenia: long-term follow-up data evaluated by the criteria of the international working group on immune thrombocytopenic purpura. Turk J Haematol (2014) 31(1):32. 10.4274/Tjh.2012.0049 24764727 PMC3996642

[5] D’Orazio J Neely J Farhoudi N. ITP in children: pathophysiology and current treatment approaches. J Pediatr Hematol Oncol (2013) 35(1):1–13. 10.1097/MPH.0b013e318271f457 23073045

[6] Lok S Kaushansky K Holly R Kuijper J Lofton-Day C Oort P Cloning and expression of murine thrombopoietin cDNA and stimulation of platelet production in vivo. Nature (1994) 369(6481):565–8. 10.1038/369565a0 8202158

[7] Yang A. Development of romiplostim: a novel engineered peptibody. Semin Hematol (2015) 52(1):12–5. 10.1053/j.seminhematol.2014.10.007 25578414

[8] Nichol J. Endogenous TPO (eTPO) levels in health and disease: possible clues for therapeutic intervention. Stem Cells (1998) 16(2):165–75. 10.1002/stem.5530160719 11012188

[9] Kuter D. The biology of thrombopoietin and thrombopoietin receptor agonists. Int J Hematol (2013) 98(1):10–23. 10.1007/s12185-013-1382-0 23821332

[10] Nichol J. AMG 531: an investigational thrombopoiesis-stimulating peptibody. Pediatr Blood Cancer (2006) 47(S5):723–5. 10.1002/pbc.20972 16933266

[11] Li C Zheng L. The pharmacology and clinical application of thrombopoietin receptor agonists. Int J Hematol (2014) 100(6):529–39. 10.1007/s12185-014-1660-5 25230815

[12] Bussel J Buchanan G Nugent D Gnarra D Bomgaars L Blanchette V A randomized, double-blind study of romiplostim to determine its safety and efficacy in children with immune thrombocytopenia. Blood (2011) 118(1):28–36. 10.1182/blood-2010-10-313908 21502541

[13] Elalfy M Abdelmaksoud A Eltonbary K. Romiplostim in children with chronic refractory ITP: randomized placebo controlled study. Ann Hematol (2011) 90(11):1341–4. 10.1007/s00277-011-1172-9 21318572

[14] Mokhtar G Tantawy A El Sherif N. Romiplostim therapy in children with unresponsive chronic immune thrombocytopenia. Platelets (2012) 23(4):264–73. 10.3109/09537104.2011.619601 22471399

[15] Vilaplana V Aragonés J Fernández-Llamazares C Bieler C Rodríguez S Sáez M. Use of romiplostim for primary immune thrombocytopenia in children. Pediatr Hematol Oncol (2012) 29(2):197–205. 10.3109/08880018.2011.629401 22376020

[16] Pasquet M Aladjidi N Guiton C Courcoux M Munzer M Auvrignon A Romiplostim in children with chronic immune thrombocytopenia (ITP): the French experience. Br J Haematol (2014) 164(2):266–71. 10.1111/bjh.12609 24152194

[17] Seidel M Urban C Sipurzynski J Beham-Shmid C Lackner H Benesch M High response rate but short-term effect of romiplostim in paediatric refractory chronic immune thrombocytopenia. Br J Haematol (2014) 165(3):419–21. 10.1111/bjh.12766 24484542

[18] Ramaswamy K Hsieh L Leven E Thompson M Nugent D Bussel J. Thrombopoietic agents for the treatment of persistent and chronic immune thrombocytopenia in children. J Pediatr (2014) 165(3):600–5. 10.1016/j.jpeds.2014.03.060 24857517

[19] Bussel J Hsieh L Buchanan G Stine K Kalpatthi R Gnarra D Long-term use of the thrombopoietin-mimetic romiplostim in children with severe chronic immune thrombocytopenia (ITP). Pediatr Blood Cancer (2014) 62(2):208–13. 10.1002/pbc.25136 25345874 PMC4309514

[20] Kuter D Bussel J Newland A Baker R Lyons R Wasser J Long-term treatment with romiplostim in patients with chronic immune thrombocytopenia: safety and efficacy. Br J Haematol (2013) 161(3):411–23. 10.1111/bjh.12260 23432528

[21] Saleh M Bussel J Cheng G Meyer O Bailey C Arning M Safety and efficacy of eltrombopag for treatment of chronic immune thrombocytopenia: results of the long-term, open-label EXTEND study. Blood (2013) 121(3):537–45. 10.1182/blood-2012-04-425512 23169778

[22] Mitchell W Bussel J. Thrombopoietin receptor agonists: a critical review. Semin Hematol (2015) 52(1):46–52. 10.1053/j.seminhematol.2014.11.001 25578419

[23] Ghanima W Geyer J Lee C Boiocchi L Imahiyerobo A Orazi A Bone marrow fibrosis in 66 patients with immune thrombocytopenia treated with thrombopoietin-receptor agonists: a single-center, long-term follow-up. Haematologica (2014) 99(5):937–44. 10.3324/haematol.2013.098921 24463212 PMC4008112

[24] Cheng G. Eltrombopag, a thrombopoietin-receptor agonist in the treatment of adult chronic immune thrombocytopenia: a review of the efficacy and safety profile. Ther Adv Hematol (2012) 3(3):155–64. 10.1177/2040620712442525 23556122 PMC3573439

